# A40 INITIAL OUTCOMES FOLLOWING THE LAUNCH OF A PERORAL ENDOSCOPIC MYOTOMY (POEM) PROGRAM FOR ACHALASIA: A SINGLE-CENTRE EXPERIENCE IN CANADA

**DOI:** 10.1093/jcag/gwae059.040

**Published:** 2025-02-10

**Authors:** C H Tsai, M Woo, M Gupta, C Schieman, P J Belletrutti

**Affiliations:** University of Calgary Cumming School of Medicine, Calgary, AB, Canada; University of Calgary Cumming School of Medicine, Calgary, AB, Canada; University of Calgary Cumming School of Medicine, Calgary, AB, Canada; University of Calgary Cumming School of Medicine, Calgary, AB, Canada; University of Calgary Cumming School of Medicine, Calgary, AB, Canada

## Abstract

**Background:**

Peroral endoscopic myotomy (POEM) has gained prominence as a minimally invasive therapeutic option for achalasia. Despite its growing adoption worldwide, the establishment of POEM programs in Canada is relatively recent, and local data on clinical outcomes remain limited.

**Aims:**

This study aims to assess the short-term outcomes of patients undergoing POEM for achalasia including symptom improvement, complication rates, and detailing the endoscopic and hospital resources necessary to sustain a POEM program.

**Methods:**

A retrospective chart review was conducted to collect procedural and clinical outcome data for patients who underwent POEM and completed follow-up assessments from the inception of the program in October 2023 to September 2024 at the Foothills Medical Centre in Calgary, Alberta. The Eckardt symptom score was used to objectively evaluate symptoms pre-operatively and one-month post-POEM.

**Results:**

A total of 20 patients, with a mean age of 59.2 (± 13.4) years, completed a one-month postoperative assessment after POEM. Each procedure was performed jointly by a gastroenterologist and a thoracic surgeon in the operating room. Most patients (16/20) were classified as ASA category II. The median Charlson Comorbidity Index was 2. 65.0% of patients had prior endoscopic interventions for achalasia, including pneumatic dilation or botulinum toxin injection. The pre-procedural mean integrated relaxation pressure at the lower esophageal sphincter was 25.8 ± 11.4 mmHg. The mean POEM procedural time was 110.5 (± 33.6) minutes, with an average of 6.5 endoscopic clips used per case for tunnel closure. The esophageal myotomy lengths were 4.79 (± 0.38) cm for type I and type II achalasia, and 9.0 (± 0.89) cm for type III achalasia. The postoperative length of stay was a median of 1 day. Postoperative complications occurred in 2 patients, both of which were managed conservatively, with no procedure-related deaths. The mean pre-POEM Eckardt score was 7.00 (± 1.79), which decreased to a mean of 1.00 (± 0.89) at one-month post-POEM (p < 0.001) [Figure 1].

**Conclusions:**

POEM is a safe and effective treatment option for patients with symptomatic achalasia. We demonstrate marked initial symptom improvement and a low complication rate. However, POEM is resource- and time-intensive. Further studies are warranted to evaluate long-term outcomes and complications such as gastroesophageal reflux disease (GERD) and Barrett’s esophagus.

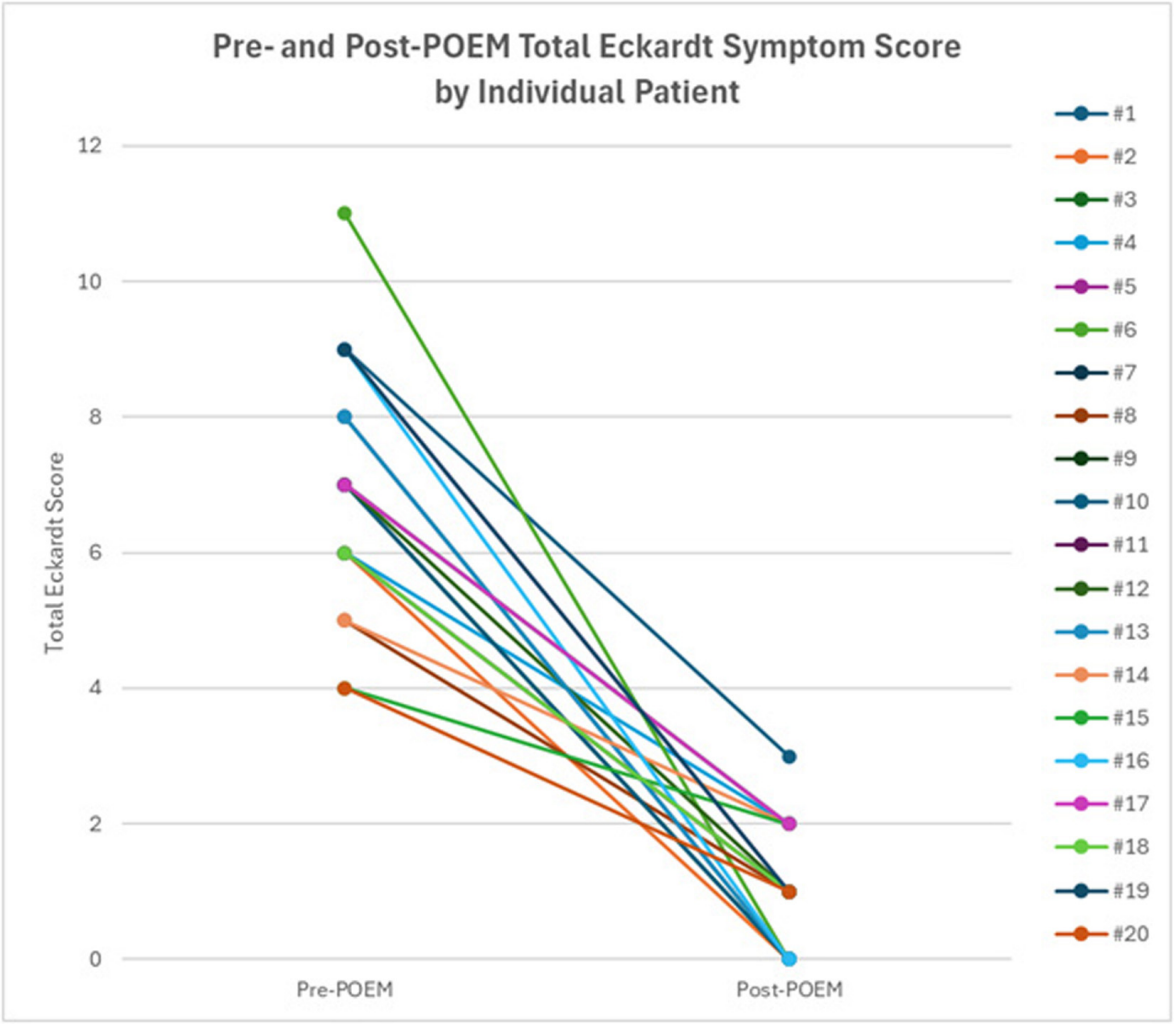

Figure 1: Total Eckardt symptom score before and after POEM. Each line represents data from an individual patient.

**Funding Agencies:**

